# Design of an Innovative Methodology for Cerebrospinal Fluid Analysis: Preliminary Results

**DOI:** 10.3390/s21113767

**Published:** 2021-05-28

**Authors:** Tommaso Schirinzi, Alberto Cordella, Nicola Biagio Mercuri, Arnaldo D’Amico, Andrea Palombi, Alessandro Zompanti, Simone Grasso, Giorgio Pennazza, Marco Santonico

**Affiliations:** 1 Department of Systems Medicine, University of Roma Tor Vergata, 00133 Rome, Italy; t.schirinzi@yahoo.com; 2 IRCCS Fondazione Santa Lucia, Via Ardeatina 306, 00179 Rome, Italy; alberto_cordella@live.it; 3 Department of Fundamental Neurosciences, University of Lausanne, 1015 Lausanne, Switzerland; 4 Department of Electronic Engineering, University of Roma Tor Vergata, 00133 Rome, Italy; damico@eln.uniroma2.it (A.D.); andreaplo89@hotmail.it (A.P.); 5 Department of Engineering, Campus Bio-Medico, University of Rome Italy, 00128 Rome, Italy; a.zompanti@unicampus.it (A.Z.); g.pennazza@unicampus.it (G.P.); 6 Department of Science and Technology for Humans and the Environment, Campus Bio-Medico, University of Rome Italy, 00128 Rome, Italy; s.grasso@unicampus.it

**Keywords:** CSF, voltammetry, electrochemical analysis, biomarkers, neurodegenerative diseases

## Abstract

Cerebrospinal fluid (CSF) analysis supports diagnosis of neurodegenerative diseases (NDs), however a number of issues limits its potentialities in clinical practice. Here, a newly developed technique for fluid voltammetry, relying on a simple sensor (BIOsensor-based multisensorial system for mimicking Nose, Tongue and Eyes, BIONOTE), was used to test the applicability for CSF analysis. BIONOTE was initially calibrated on an artificial CSF-like solution and then applied on human CSF, either immediately after collection or after refrigerated storage. Following optimization, it was used to evaluate 11 CSF samples correlating the electrochemical dataset with CSF routine parameters and biomarkers of neurodegeneration. Multivariate data analysis was performed for model elaboration and calibration using principal component analysis and partial least squares discriminant analysis. BIONOTE presented a high capacity to predict both physiological and pathological constituents of artificial CSF. It differentiated distinct fresh human CSF samples well but lost accuracy after refrigerated storage. The electrochemical analysis-derived data correlated with either CSF routine cytochemical indexes or a biomarker of neurodegeneration. BIONOTE resulted as being a reliable system for electrochemical analysis of CSF. The CSF fingerprint provided by the sensor has shown itself to be sensitive to CSF modification, thus it is potentially representative of CSF alteration. This result opens the way to its testing in further study addressed at assessing the clinical relevance of the methodology. Because of its advantages due to the ease and rapidity of the methodology, a validation study is now required to translate the technique into clinical practice and improve diagnostic workup of NDs.

## 1. Introduction

Neurodegenerative diseases (NDs) are a heterogeneous group of progressive and disabling conditions whose worldwide incidence and prevalence are increasing following global aging and longer life expectancy.

Neuropathology and pathophysiology differ among NDs; conversely, clinical disturbances, including both motor and cognitive disturbances, may overlap. Indeed, while Alzheimer’s disease (AD) and Parkinson’s disease (PD), the two most common NDs, basically present with typical syndromes, there are many other cases in which the diagnosis is more challenging because of the clinical complexity and variety [1].

Nowadays NDs remain incurable disorders. However, the therapeutic scenario is rapidly changing and potential disease-modifying treatments are upcoming. Therefore, the diagnostic certainty now represents an urgent need in the field of NDs, since the therapeutic success may depend on a timely and precise diagnosis [2,3].

To date, the diagnosis of different NDs mostly relies on clinical criteria, whereas instrumental findings (e.g., neuroimaging or fluid biomarkers) offer support. Actually, fluid biomarkers are constantly gaining more relevance, such that cerebrospinal fluid (CSF) biomarkers are crucial in diagnosis and stratification of patients with suspected AD, as well as allowing prognostic evaluations in patients with PD [3,4,5,6].

CSF, indeed, reflects pathological changes occurring at the central nervous system (CNS) level, thus representing a valuable source for reliable biomarkers informing on the biological events underlying clinical conditions. Despite these theoretical advantages, development and use of CSF biomarkers are still limited by several factors, which include either the scarcity of molecular targets or issues related to the validation and spread of assay methodologies. In this perspective, the application of novel technologies in CSF analysis aimed at overcoming current barriers would be fundamental [7].

In this direction, here we present a newly developed technique based on voltammetry oriented towards a detailed chemical description of fluids’ characteristics [8,9].

This technique is based on a sensor system able to analyze many different biological fluids [8,9], already tested in numerous experiments in the medical field [10,11,12,13].

The method of the analysis of CSF is not new, and this analyte has been also measured with non-conventional sensors (meaning not routine clinical instruments) [14,15,16,17] but this analysis has never been performed with an electrochemical sensor using a fingerprinting approach. Moreover, the novelty not only consists in using a diverse instrument but in using an instrument with a series of characteristics offering innovative potentialities to obtain (after further developments) easier, faster and cheaper methodologies with respect to the existing ones.

In particular, we preliminarily applied the technique to artificial CSF in order to assess applicability and performance in terms of sensitivity and resolution.

Then we tested this technique on samples of human-derived CSF, in order to evaluate its potentialities and to understand its relevance for future use in clinical frames.

Considering the simplicity of the instrument, the low costs of each measurement and the fingerprinting approach (not selective for the single compound, but sensitive to a spectrum of elements and factors), the goal of this work consists in demonstrating the feasibility of the application via a proof of concept experiment. 

## 2. Materials and Methods

### 2.1. Voltammetric System and Experimental Design

The voltammetric sensor consists of a sensing platform, named BIONOTE (BIOsensor-based multisensorial system for mimicking Nose, Tongue and Eyes [8,9]), including a screen-printed electrode (SPE; DRP-250BT, Metrohm, Herisau, Switzerland) probe and a dedicated electronic interface providing an input signal and recording the output data. The SPE composition is the following: the working electrode is made of gold, the counter electrode is made of platinum, the reference electrode is made of silver and all the other electric contacts are made of silver. The signal input consists of a triangular waveform between −1 V and 1 V and a frequency of 0.01 Hz. When the sensor probe is immersed in a solution, a current related to the oxy-reductive reactions occurring in the sample is recorded. This current is the output signal response of the sensor, and it is converted into a voltage value by a trans-impedance circuit. The system is composed of an Analogo to Digital Converter (ADC) able to guarantee a sampling rate of 200 ms. During each period, we can collect 500 output values for each measuring cycle. The entire dataset is then treated as a global electrochemical signature (composed of 500 points) of the analyzed sample.

This instrument has been already used with human fluids, namely for etiological diagnosis of pleural effusion [10], ascites identification based on serum-ascites analysis [11], and lower-limb ulcers monitoring based on exudate analysis [12].

Since BIONOTE has never been tested on CSF, here we set an experimental plane aiming to: (1) estimate BIONOTE sensitivity and resolution in the assessment of CSF samples such to be used for the content analysis and between-samples discrimination; (2) assess the reliability and effectiveness of BIONOTE-based analysis after CSF sample storage; (3) evaluate the capability of BIONOTE to differentiate CSF samples from different clinical conditions.

Aim 1 has been addressed by an experiment of calibration of the BIONOTE to the artificial CSF constituents; aim 2 has been addressed by the optimization of the experimental setup for human CSF analysis; aim 3 has been addressed by applying the BIONOTE analysis protocol on a small number of patient-derived CSF samples as a pilot experience.

### 2.2. Preparation of the Standard Curves for Calibration Purposes

All standard solutions used for calibration purposes were prepared dissolving the chemical compound in bi-distilled water at the higher concentration and by following dilutions. All chemical standards were purchased at Sigma Aldrich (Merck KGaA, Darmstadt, Germany): potassium chloride (P3911), sodium chloride (S7653), sodium bicarbonate (S5761), calcium chloride (C5670), magnesium chloride (M8266), Sodium hydrosulfide hydrate (161527), dopamine hydrochloride (H8502). All these samples were prepared and measured in glass test-tubes.

The BIONOTE was initially challenged against these CSF constituents individually as described in [13]. Seven calibration curves were prepared using the chemical standard compounds reported in Table 1.

### 2.3. Preparation of the Artificial Cerebrospinal Fluid (CSF)

To find further evidence supporting the employability of the BIONOTE system for CSF analysis, an artificial CSF was prepared.

The artificial CSF was freshly prepared before each experimental session dissolving in bi-distilled water the compounds reported in Table 2 [18,19].

Next, dopamine and NaHS were added at different concentrations, mimicking some alterations occurring in NDs [20] (Table 3).

To prevent undesirable oxidation phenomena of dopamine, which may potentially affect the voltammetric analysis, each solution was bubbled with nitrogen before dopamine addition [8].

### 2.4. Human CSF Sampling

CSF samples were obtained from a total of 11 subjects (36% females) undergoing lumbar puncture (LP) for diagnostic purpose at the Neurology Unit of Tor Vergata University Hospital (Rome, Italy). None of them was diagnosed with NDs (basically affected by headache, psychogenic disorders or peripheral nervous system disorders). 

LP was performed at the bedside, in the morning, after an overnight fast, with the subject lying in lateral decubitus position. CSF samples were collected and processed according to standard procedures. Analysis was carried at the local laboratory and included routine quantitative assessment of glucose, lactate, total proteins, albumin, white blood cells, Immunoglobulin G (IgG), and the evaluation of neurodegeneration-related biomarkers amyloid-β-42 peptide, total tau protein, 181-phosphorylated tau protein [5,21] (Table 4).

Five of these 11 samples were further used for a repeatability evaluation by being measured multiple times immediately after the lumbar puncture, and a reproducibility estimation over time by being re-analysed after 30 and 120 days of stocking at −80 °C.

### 2.5. Data Analysis

The electrochemical pattern registered by the voltammetric sensor holds a multidimensional (500 points) and enriched informative content. The complex nature of this dataset required the application of multivariate data analysis techniques to provide a simplified representation of the multidimensional space acquired and to highlight the most informative features.

Principal components analysis (PCA) allowed the representation of the multidimensional dataset onto a simplified scatter chart by defining the most important variables that characterized the specific phenomenon of interest [22].

Each measurement of the data set is represented by a point on the PCA plane (the score plot of the most significant principal components). When the points are close one to each other, they form a cluster of samples with similar characteristics. The points far from this cluster have different characteristics.

Calibration was performed via partial least squares discriminant analysis (PLS-DA) coupled with the leave-one-out criterion as a cross-validation method in order to obtain all the predictive models on the calibration data.

All the multivariate data analyses were performed using PLSToolbox (Eigenvector Research Inc., Manson, WA, USA) in Matlab Environment (The MathWorks, Natick, MA, USA).

The fingerprints obtained by the sensor reflected the chemical composition of the measured samples. Thus, a multidimensional model was elaborated via PLS-DA using the leave-one-out criterion; this model received as input the multidimensional fingerprint and is able to predict with a reasonable error (root mean square error in cross validation) the concentration of the different compounds in the sample solution.

**Figure 1:**
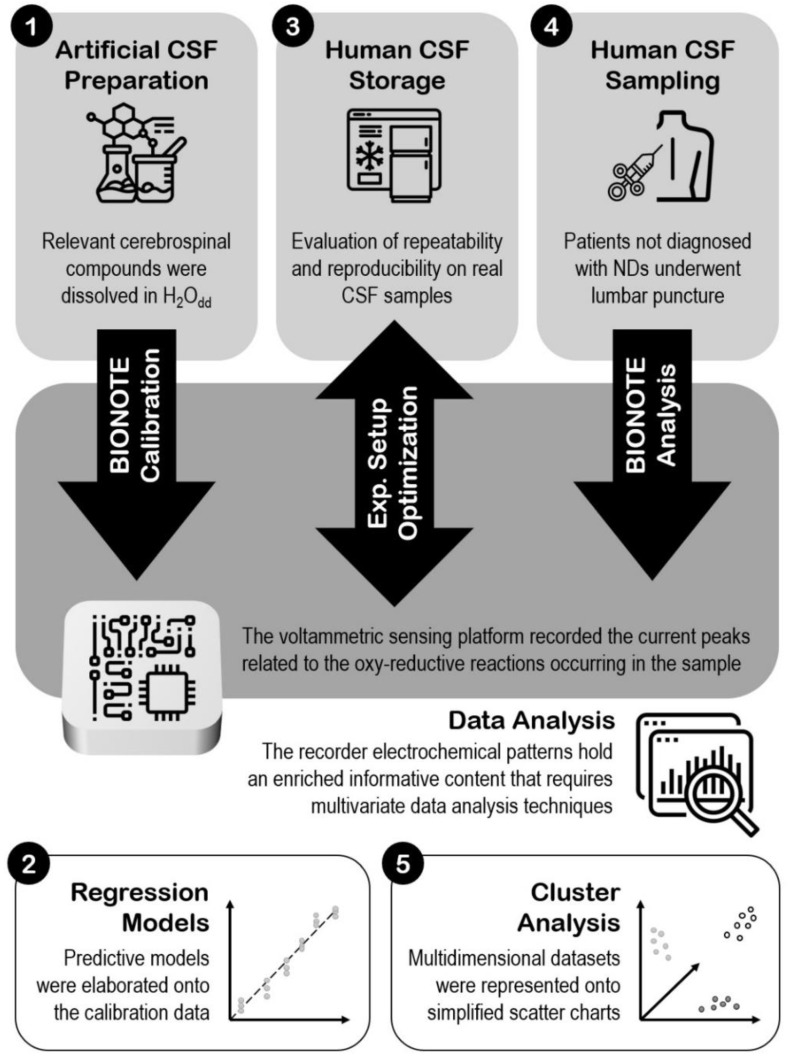
Experimental set-up.

Figure 1 resumes the whole steps of the work, giving an overview of the experimental set-up.

## 3. Results

### 3.1. Calibration of the BIONOTE (Biosensor-Based Multisensorial System for Mimicking Nose, Tongue and Eyes) to the Single Artificial CSF Constituents

**Figure 2:**
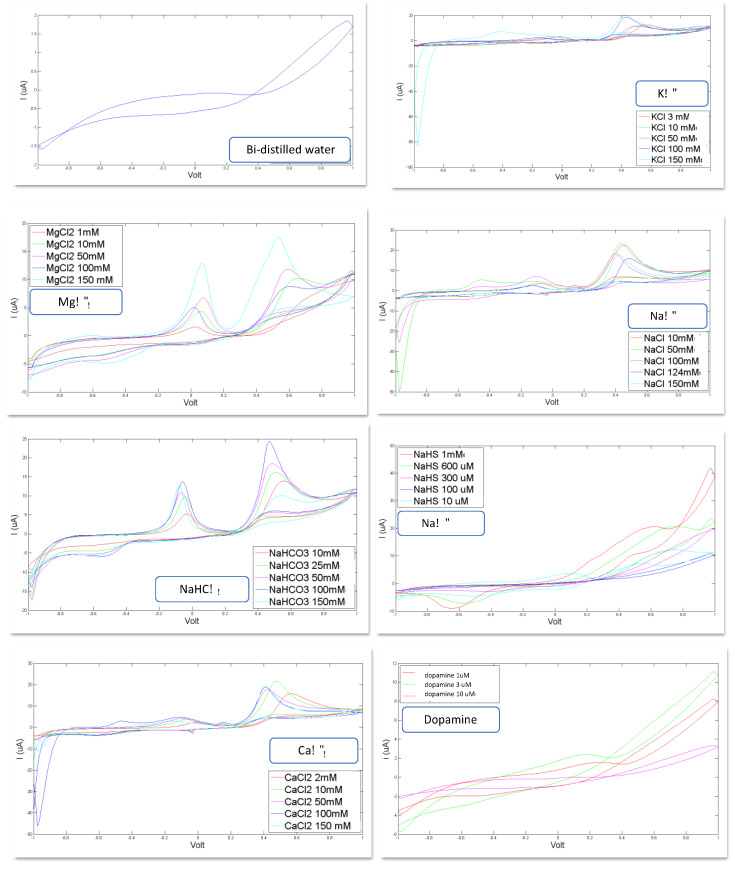
Voltammograms registered for the following constituents for different concentration levels: reference bi-distilled water KCl (3, 10, 50, 100, 150 mM, MgCl_2_ (1, 10, 50, 100, 150 mM), NaCl (10, 50, 100, 124, 150 mM), NaHCO_3_ (10, 25, 50, 100, 150 mM), NaHS (10 µM, 100 µM, 300 µM, 600 µM, 1 mM), CaCl_2_ (2, 10, 50, 100, 150 mM), Dopamine (1 µM, 3 µM, 10 µM).

In Figure 2, the voltammograms registered for each of the eight artificial CSF constituents are shown. Bi-distilled water is the reference standard used for the calibration of each compound and as basis solute for the artificial CSF: KCl, MgCl_2_, NaCl, NaHCO_3_, CaCl_2,_ NaHS and dopamine dissolved in bi-distilled water.

Important confirmations can be inferred from the curves shown in Figure 2: these curves corroborate the feasibility of the study because they show the ability of the sensor system to recognize the constituents of the CSF and to distinguish different concentration levels. 

Indeed, it is evident that each compound generates a voltammogram with a peculiar profile; each of these voltammograms is different, in shape, from the others; these profiles preserve their shape for each constituent when varying the concentration level, but this shapes increase their area and the height of each peak as the concentration increases.

All these data have been used for calibration purposes. To this scope each single curve has been treated as a multidimensional data source (the 500 points acquired to form the voltammogram).

PLS-DA was used for the calibration model. The ability of the sensor inferred by the qualitative observation of the curves (above introduced) is confirmed by the encouraging values calculated for the root mean square errors in calibration (RMSEC) and in cross validation (RMSECV), for each of the constituents the artificial CSF. All these parameters are reported in Table 5. This shows the ability of BIONOTE to efficiently predict relevant CSF constituents when challenged in a condition of limited complexity.

In order to understand the influence of these RMSECV when assessing the concentration of the different compounds in bi-distilled water, the percentage error can be calculated taking into account the span of the all range of concentrations considered in the calibration measurements.

The percentage errors are the following: 6% for KCl, 1% for NaCl, 5% for NaHCO_3_, 6% for CaCl_2_, 5% for MgCl_2_, 9% for NaHS, 18% for dopamine. These percentage errors should not be affordable when the sensor is used as an analytical method for constituent quantification. In this case the goal is not quantification. The important result coming from these limited percentage errors is that the sensor is able to distinguish the modification of the artificial CSF when its composition is modified. Aiming at discriminating alteration of CSF due to a diseased condition, this is a promising result, encouraging the next step of verification, consisting in measuring artificial CSF as a whole.

### 3.2. Measurements of Artificial CSF and Their Modifications

**Figure 3:**
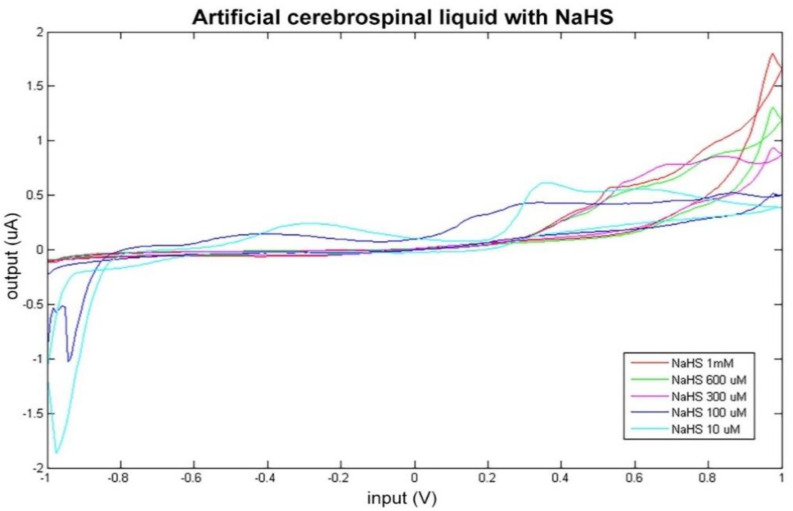
Cyclic voltammetry fingerprint of artificial cerebrospinal liquid with progressive NaHS addition.

**Figure 4:**
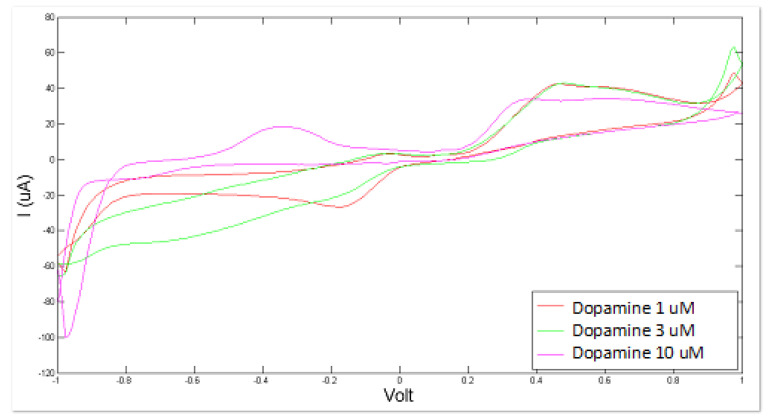
Cyclic voltammetry fingerprint of artificial cerebrospinal liquid with progressive dopamine addition.

Artificial CSF was measured as a whole in different altered conditions, simulated by varying the concentration levels of NaHS and of dopamine. The consequent modifications of the sensor response can be observed in Figure 3 and Figure 4. 

The data obtained by BIONOTE-based analysis of the artificial CSF solutions modified with dopamine and NaHS addition were elaborated through multivariate data analysis techniques (PLS) in order to mathematically confirm this ability of the sensor in discriminating altered conditions of artificial CSF. PLS regression model on NaHS modified solutions confirmed the BIONOTE’s ability to predict NaHS concentration with a RMSECV of about 0.07 mM, even in a complex mixture and with better performance with respect to NaHS as sole compound in bi-distilled water (which was 0.09 mM).

This result corroborates what arose in the comparison between the electrochemical fingerprint of the native artificial CSF and the modified one (Figure 3).

Similarly, the BIONOTE showed a satisfactory and promising performance in the analysis of dopamine-modified artificial CSF with a RMSECV of about 0.67 × 10^−3^ mM. The optimized experimental setup allowed us to achieve better performance by decreasing the error of the PLS regression model with respect to the 1.80 × 10^−3^ mM error obtained for dopamine as sole compound in bi-distilled water.

### 3.3. Optimization of the Experimental Setup for Human CSF Analysis

Once the ability to detect relevant markers in an artificial complex mixture was confirmed, the voltammetric sensor was challenged with human CSF.

The first test on human CSF samples was oriented to the optimization of the measurement protocol. Indeed, in the application of this new methodology in the field, some practical problems (not present in the lab set-up used for artificial CSF) were encountered.

The first problem was relative to the clinical practicability of the method. Indeed, it is useful to understand whether the CSF extracted from an individual must be measured immediately after extraction or can be stored and measured later. This is a problem of repeatability.

**Figure 5:**
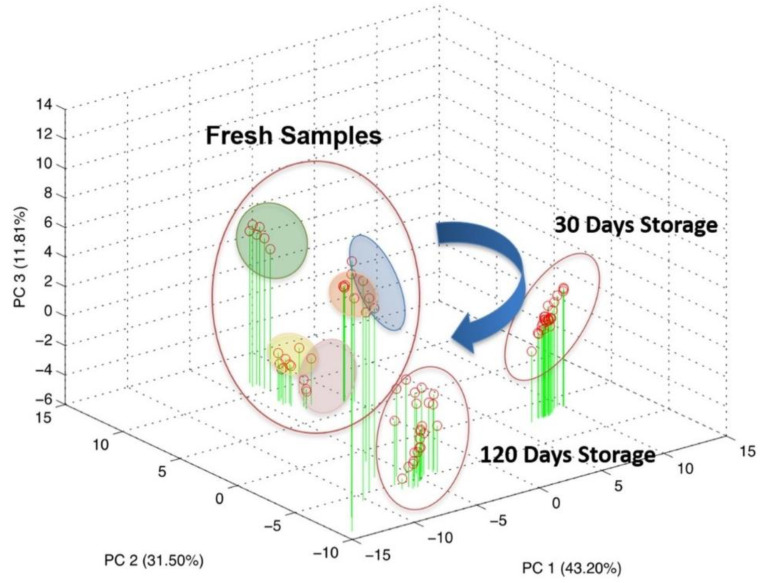
Scores plot of the principal component analysis on the overall dataset comprising both repeatability and reproducibility evaluation.

Five CSF samples, extracted from five patients, were used for a repeatability evaluation. These samples were measured multiple times immediately after the LP, and a reproducibility estimation over the time were performed via successive re-analyses of the same samples after 30 and 120 days of stocking at −80 °C. The overall dataset was elaborated through PCA, an unsupervised multivariate data analysis technique, and the result is reported in Figure 5.

As can be seen from the score plot of the PCA, the three experimental sessions shaped distinct clusters in the three-dimensional space defined by PC1, PC2 and PC3 accounting for about 86% of the explained variance.

This result highlighted the evolution of the biological sample over time, even if properly stocked at −80 °C. Particularly, applying PCA analysis over the three experimental session independently, it becomes clear that most of the information pertained to the fresh CSF tends to disappear during the storage as a result of a progressive modification.

Indeed, looking at Figure 5, it is clear that the measurements relative to the fresh samples can be clustered for each patient and each cluster is sufficiently distinguishable from each other. The clusters have been graphically identified by grouping all the repeated measurements executed for the fresh samples of the same patients. It is evident that all the points relative to the samples measured after storage of 30 days and of 120 days cannot be grouped in different clusters and are instead crunched in a cloud of overlapped points. It could be observed that this distribution of the points is due to the 3-D graphical representation. To better investigate this issue, the planes of the first two PCs (PC1 and PC2) have also been shown.

**Figure 6:**
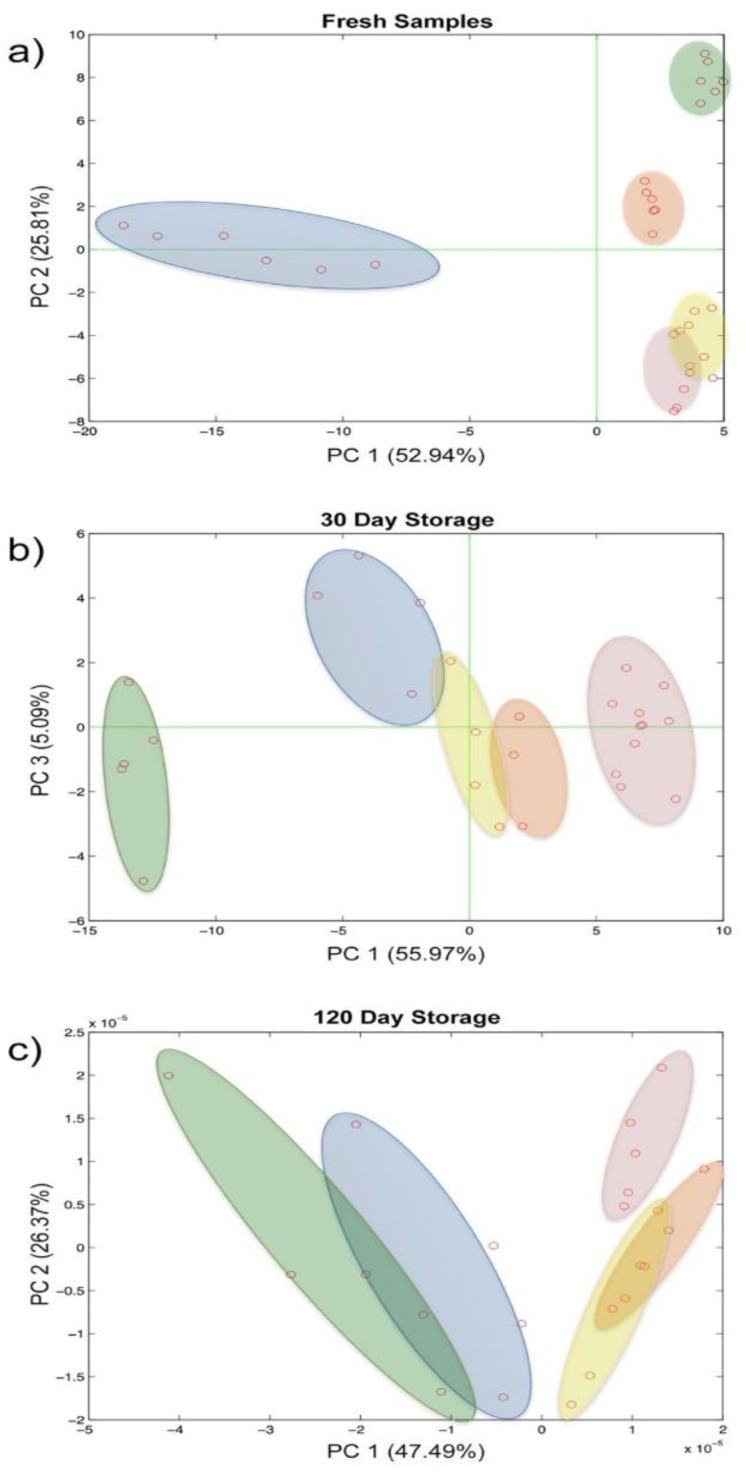
Score plot of the principal component analysis on repeatability and reproducibility evaluation: (**a**) fresh samples, (**b**) 30 days storage, (**c**) 120 days storage.

While the fresh samples were clearly distinguishable over the plane along both the PC1 and PC2, these were less divergent already after 30 days of storage and became overlapped in the last time step evaluated (Figure 6a–c). Furthermore, the repeatability of the analytical system also seems to be affected in a certain way by the evolution of the biological sample.

### 3.4. Preliminary Tests on Human CSF Samples

Eleven CSF samples from non-ND patients were analyzed with the BIONOTE voltammetric sensor immediately after LP. 

**Figure 7:**
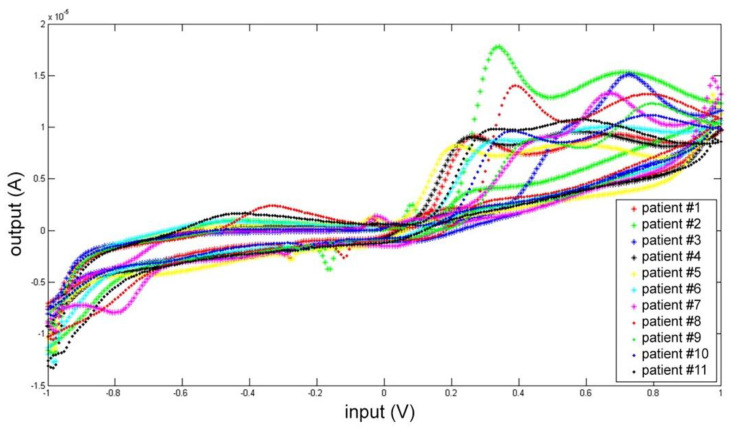
Cyclic voltammetry fingerprint of human CSF samples of the 11 patients enrolled overlapped in the same graph.

**Figure 8:**
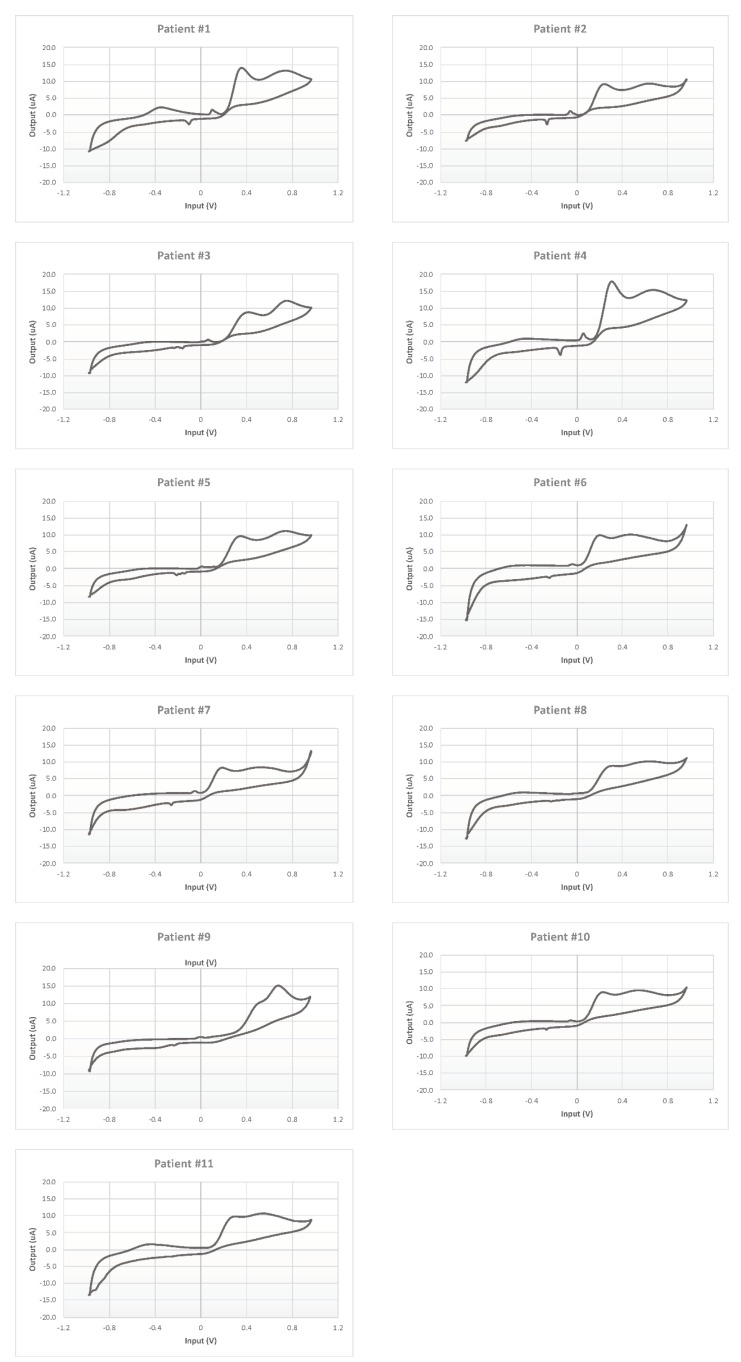
Cyclic voltammetry fingerprints of human CSF samples of each patient enrolled.

Each CSF was measured five times, and the mean electrochemical fingerprints over the five cycles have been calculated and represented in two figures: all the voltammograms together, overlapped in the same graph (Figure 7); and a graph for each single patient (Figure 8).

In Figure 7 all the voltammogram can be compared immediately. This comparison highlights a common pattern resembling the shape of the artificial cerebrospinal liquid signal (check Figure 3 and Figure 4).

Moreover, each voltammogram is characterized by peculiar peaks, probably associated with the expected interindividual variability. This behavior is put in evidence more clearly by the 11 panels of Figure 8 showing the 11 profiles of the mean responses calculated for each individual.

The BIONOTE is proposed here as a fast, easy and cheap instrument to discriminate altered CSFs against controls and, hopefully, also to discriminate CSFs referring to different diseases. This is an ambitious end-point, which can be achieved through sensor response characterization (as described above) but also comparing BIONOTE outputs against the clinical standard data. A list of biochemical parameters was selected for this test: glucose, lactate, total proteins, albumin, white blood cells, immunoglobulin-G, amyloid-β-42, total-tau, phosphorylated-181-tau.

To train the BIONOTE system to predict biochemical parameters usually retrieved by standard analytical techniques, the whole dataset was elaborated several times through multivariate data analysis against each marker. The results obtained from the PLS regression models built on the related clinical data are reported in Table 6.

Thus far, the BIONOTE showed a quite different performance ranging from optimal (glucose, total proteins, white blood cells, albumin and total-tau protein), to sub-optimal (immunoglobulin-G and phosphorylated-181-tau protein) and also inadequate (lactate and amyloid-β-42 protein).

## 4. Discussion

BIONOTE is a sensor for voltammetric analysis of fluids which has been used already for clinical applications with promising results. Indeed, it has been tested to analyze pleural, ascitic, lower-limb ulcers liquids, and urine [11,12,13]. Here, instead, it was studied for the first time with CSF, the liquid in which the CNS is immersed, whose analysis is critical to diagnose neurological disorders, including NDs. 

In this study, we initially assessed the BIONOTE performances on an artificial CSF sample to establish its capability to recognize main different physiological and pathological CSF constituents coherently with their concentration.

After such a calibration, the BIONOTE was used with human CSF obtained from five patients receiving LP for diagnostic purposes. The test was conducted in three conditions reflecting what commonly occurs in clinical practice, where CSF could be analyzed either immediately after the LP or after refrigerated storage (−80 °C). Namely, the BIONOTE-based analysis was performed repeatedly on fresh samples and after two periods of freezing, for 30 and 120 days, respectively, showing that the accuracy to differentiate distinct samples was better in fresh conditions, while it decreased progressively with −80 °C storage, probably because of CSF’s electrochemical modifications due to prolonged refrigeration.

Finally, the BIONOTE-based analysis was applied to fresh CSF samples from 11 human subjects to predict different biochemical markers. Of interest, the strongest associations resulted with routine parameters such as glucose, total proteins, white blood cells, albumin, and especially with total-tau protein, a marker of neuronal injury whose CSF levels proportionally increase with axonal degeneration in neurons, as occurs in AD [19], prion diseases, and other acute neurological disorders (e.g., status epilepticus) [20]. Correlations with more specific pathology-associated biomarkers (phosphorylated-181-tau and amyloid-β-42 protein) were, instead, weaker. This latter finding is consistent with the enrolment of subjects not diagnosed with NDs, since it is expected that phosphorylated-181-tau and amyloid-β-42 were within an established range of normality [23,24,25,26]; other markers conversely are affected by a number of conditions (e.g., age, systemic disorders), which allows different distributions of values among the individuals. 

Despite the limitation of this pilot study, such as the sample size and enrolment of a so-called “control population” (patients not diagnosed with NDs), BIONOTE emerges as a reliable device for voltammetric analysis applicable for clinical purposes even in neurology. Actually, the sensor was able to detect differences in fresh CSF due to various concentrations of both physiological and pathological constituents, which may reflect different clinical conditions. The main quality of BIONOTE is the ease and the rapidity of electrochemical analysis, which applied on fresh CSF samples may inform immediately on relevant clinical events. Indeed, electrochemical analysis relies on a simple and manageable device whose outcome is provided in a short time.

These strengths seem to be fundamental to fill current gaps of CSF-assisted diagnosis in the field of NDs. Accordingly, is now necessary to proceed with confirmatory studies involving patients on larger scale.

## 5. Conclusions

The endpoint of this work is the proof of concept of the BIONOTE as a relevant sensor for the characterization of CSF, oriented to its utilization as a fast pre-screening instrument to address further analysis and therapies. This clinical use is beyond the scope of the work, while the proof of concept has been shown by the pilot study presented. BIONOTE output is characteristic and reproducible for artificial CSF and for the human CSF. Thus, the methodology developed and tested is effective and its application shown a good reproducibility. Moreover, the CSF fingerprint provided by the sensor has been shown to be sensitive to CSF modification, and thus it is potentially representative of CSF alteration. This result opens the way to its testing in a further study addressed to assess the clinical relevance of the methodology.

## Figures and Tables

**Table 1:** Calibration curve schema of relevant cerebrospinal liquid compounds.

Chemical Standard Concentration (mM)							
**KCl**	3	10	50	100		150	
**NaCl**	10	50	100	124		150	
**NaHCO_3_**	10	25	50	100		150	
**CaCl_2_**	2	10	50	100		150	
**MgCl_2_**	1	10	50	100		150	
**NaHS**	0.01	0.1	0.3	0.6		1	
**Dopamine**	0.001	0.003	0.01		
			

**Table 2:** Chemical composition of the artificial cerebrospinal liquid.

	KCl	NaCl	NaHCO_3_	CaCl_2_	MgCl_2_	NaHS
**mM**	3	124	26	2	1	1.25

**Table 3:** Artificial modified-cerebrospinal fluid (CSF) standard concentration (mM) at different concentrations.

Chemical Standard Concentration (mM)					
**NaHS**	0.01	0.1	0.3	0.6	1
**Dopamine**	0.001	0.003	0.005	0.007	0.01

**Table 4:** Clinical and demographic data of the study population.

Variable	Mean	st.dev.
Age (years)	62	13.8
Glucose (mg/dL)	57.1	4.7
Lactate (mmol/L)	1.8	0.2
Total proteins (mg/dL)	48.3	19.4
Albumin (mg/dL)	28.4	16.1
White blood cells (mmc)	3.1	2.4
Immunoglobulin-G (mg/dL)	5.7	7.6
Amyloid-β-42 (pg/mL)	715.4	378.8
Total-tau (pg/mL)	340.3	165.9
Phosphorylated-181-tau (pg/mL)	83	96.1

**Table 5:** Partial least squares regression: root mean square error in calibration (RMSEC) and root mean square error in cross-validation (RMSECV) expressed as mM concentration.

	KCl	NaCl	NaHCO_3_	CaCl_2_	MgCl_2_	NaHS	Dopamine
**RMSEC**	3.62	1.39	0.09	0.43	0.13	0.84 × 10^−2^	0.02 × 10^−3^
**RMSECV**	9.69	11.01	8.01	8.66	7.89	9.26 × 10^−2^	1.80 × 10^−3^

**Table 6:** Partial least squares regression: root mean square error in calibration (RMSEC) and root mean square error in cross-validation (RMSECV) of CSF biochemical markers.

	RMSEC	RMSECV
**Glucose (mg/dL)**	0.15	4.28
**Lactate (mmol/L)**	0.18	0.29
**Total proteins (mg/dL)**	0.37	19.93
**Albumin (mg/dL)**	0.76	16.53
**White blood cells (mmc)**	0.69	2.53
**Immunoglobulin-G (mg/dL)**	0.62	2.21
**Amyloid-β-42** **(pg/mL)**	275.21	510.25
**Total-tau (pg/mL)**	0.90	176.39
**Phosphorylated-181-tau (pg/mL)**	30.19	120.94

## Data Availability

Data are available upon reasonable request.
